# Transient Complete Unilateral Oculomotor Nerve Palsy following Clipping of Ruptured Anterior Communicating Artery Aneurysm: An Abstruse Phenomenon

**DOI:** 10.1155/2019/3185023

**Published:** 2019-02-05

**Authors:** Joe M. Das, Rashmi Sapkota, Manish Mishra

**Affiliations:** ^1^Consultant Neurosurgeon, Department of Neurosurgery, College of Medical Sciences–Teaching Hospital, Bharatpur–10, Chitwan, Nepal; ^2^Sister-in-Charge, Department of Neurosurgery, College of Medical Sciences–Teaching Hospital, Bharatpur–10, Chitwan, Nepal; ^3^Medical Officer, Department of Neurosurgery, College of Medical Sciences–Teaching Hospital, Bharatpur–10, Chitwan, Nepal

## Abstract

**Background:**

Aneurysmal subarachnoid hemorrhage may be associated with different cranial nerve palsies, with oculomotor nerve palsy (ONP) being the most common. ONP is especially associated with posterior communicating artery aneurysms, due to the anatomical proximity of the nerve to the aneurysmal wall. Anterior communicating artery (Acom) aneurysms are very unlikely to produce ONP due to the widely separated anatomical locations of Acom and oculomotor nerve.

**Case Description:**

Here we describe the case of a 60-year-old nondiabetic lady who presented with Acom aneurysmal subarachnoid hemorrhage having a World Federation of Neurosurgical Societies (WFNS) grade I. She underwent an uneventful right pterional craniotomy and clipping of the aneurysm, except for a short period of controlled rupture of the aneurysm. Postoperatively she developed complete ONP on the right side, though her sensorium was preserved. Computed Tomogram and Magnetic Resonance Imaging scans of the brain did not yield any useful information regarding its etiology. She was conservatively managed and kept on regular follow-up. She had a gradual recovery of ONP in the following order: pupillary reaction, ocular movements, and finally ptosis. On postoperative day 61, she had complete recovery from ONP.

**Conclusion:**

We describe a very unusual case of complete ONP following Acom aneurysm clipping and its management by masterly inactivity.

## 1. Introduction

“Perhaps the most important evidence of aneurysm is the sudden repeated sharp pains in the eye or in the frontal or temporal region, frequently followed by ptosis of the upper lid and extraocular palsies on the same side. These manifestations are almost pathognomonic of carotid or nearby aneurysms.” (Dandy WE, 1944) [1].

This holds true even now. In any patient presenting with spontaneous painful oculomotor nerve palsy (ONP), intracranial aneurysm has to be suspected, especially that located in the posterior communicating, cavernous internal carotid, basilar, posterior cerebral, anterior choroidal, or superior cerebellar arteries [2]. In these situations, it is easy to explain the presence of ONP due to the close proximity of oculomotor nerve to the above-said aneurysms. The aneurysm, once it enlarges, can compress on the nerve producing pain and palsy. The other causes of ONP in patients with aneurysmal subarachnoid hemorrhage (aSAH) are transtentorial herniation, iatrogenic injury, or associated diabetes mellitus. We encountered a patient who developed a painful ophthalmoplegia, following surgical clipping of ruptured anterior communicating artery (Acom) aneurysm. Though rarely reported to produce both abducens and trochlear nerve palsies [3], Acom aneurysm is very unlikely to produce ONP (with only 11 such cases reported till date in the literature) due to the anatomically remote locations of the two structures.

## 2. Case Report

A 60-year-old lady, who was a hypertensive patient under irregular medication, presented with mild-to-moderately-severe headache episodes for four days for which she did not seek medical attention. This was followed by sudden onset severe headache for one day prior to presentation in our emergency room (ER). Headache was holocranial and associated with vomiting. There was no history of trauma, fever, seizures, weakness of limbs, or loss of consciousness. She was not a diabetic and did not have any addictions.

**Figure 1:**
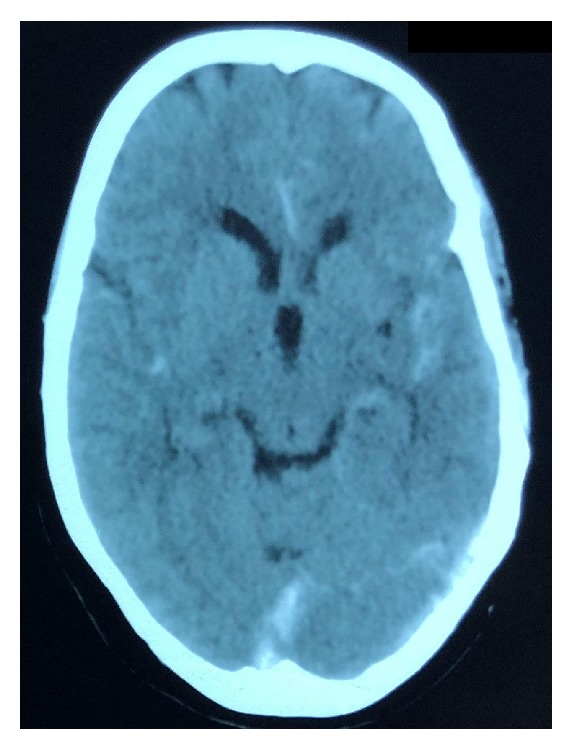
Preoperative plain Computed Tomogram scan of the brain showing subarachnoid hemorrhage.

When she presented to our ER, her Glasgow Coma Scale score was 15 and did not have any neurological deficits (World Federation of Neurosurgical Societies grade I). She underwent plain Computed Tomogram (CT) scan of the brain, which showed subarachnoid hemorrhage (SAH) in the left sylvian fissure and interhemispheric fissure (Modified Fisher grade 1) (Figure 1). Suspecting an aneurysmal SAH, she was admitted in neurosurgery intensive care unit and was started on antiedema measures, anticonvulsant, analgesic, and Nimodipine.

**Figure 2:**
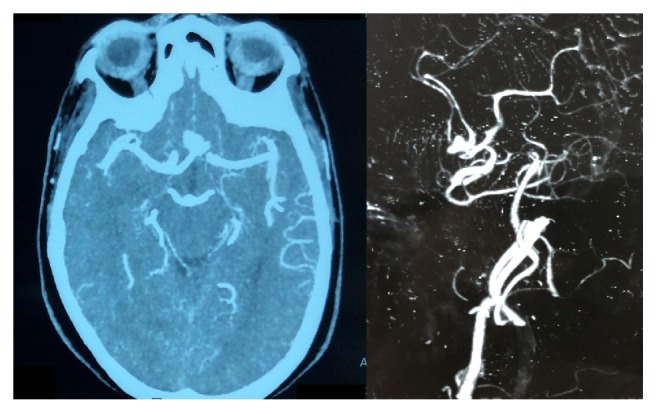
Computed Tomogram Angiography films showing the anterior communicating artery aneurysm.

The next day, she underwent CT cerebral angiogram, which revealed a bilobed anterior communicating artery aneurysm, projecting anterosuperiorly and measuring 8 × 7 × 5 mm in size (Figure 2). There was no evidence of any other aneurysms or vascular malformations. On the fourth day of ictus, she underwent right pterional craniotomy and clipping of aneurysm.

Intraoperatively, the sphenoid drilling and craniotomy were uneventful. After exposure of the aneurysm, there was controlled rupture during permanent clipping with a blood loss of around 20 ml and temporary clipping was not required. Papaverine was not instilled. Since the brain was slightly full at the end of surgery, the bone flap was not replaced. She was extubated postoperatively on table and was fully conscious.

**Figure 3:**
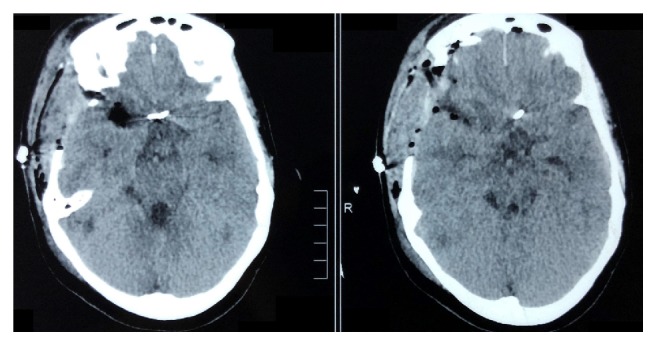
Postoperative plain Computed Tomogram scan of the brain showing the absence of hematoma or infarct.

**Figure 4:**
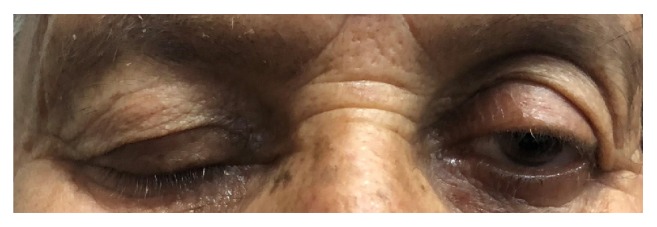
Photograph showing the right sided ptosis due to oculomotor nerve palsy.

**Figure 5:**
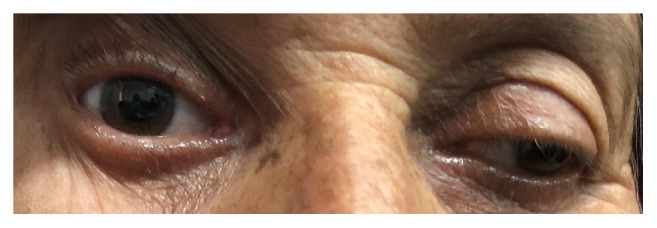
Photograph showing the manually lifted right eye lid demonstrating mydriasis and impaired adduction of right eye.

**Figure 6:**
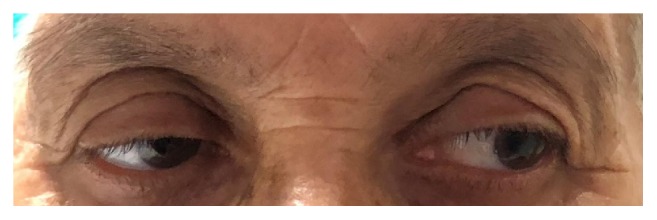
Photograph showing the resolved oculomotor palsy with full adduction of right eye.

Three hours after the surgery, she started developing right sided ptosis, which progressed into complete right sided oculomotor nerve paralysis with dilated and nonreacting pupil. An emergency CT scan of the brain was taken which revealed only postoperative changes (Figure 3). There was no hematoma in the basal cisterns or infarct. But her oculomotor nerve palsy persisted and was painful (Figure 4). Her further postoperative period was uneventful, pupillary reaction to light started to appear, and pain started to disappear by day 7. But pupillary size remained the same (Figure 5). She was discharged on the eighth postoperative day. On follow-up after one week, a Magnetic Resonance Imaging scan of the brain with venogram was done to rule out any infarct or thrombosis of the cavernous sinus. But it turned out to be normal. She was kept under regular follow-up in our outpatient department. Nimodipine was continued for a total of 21 days following the ictus. On review at the end of one month, her ocular movements were normal except for impaired adduction and pupils were normal in size and reaction, but complete ptosis was persisting. On the 61st postoperative day, her ptosis suddenly disappeared on waking up and when she came for follow-up in outpatient department, her ONP had fully recovered (Figure 6).

## 3. Discussion

Oculomotor nerve is the most common cranial nerve affected in patients with aneurysmal subarachnoid hemorrhage [11]. Depending on the series, 15% to 50% of oculomotor nerve palsies (ONP) are caused by an intracranial aneurysm [10] and paresis of oculomotor nerve is associated with ruptured aneurysms in 30% of patients [12]. Aneurysmal ONP typically presents with pain, mid-dilated pupil with poor or absent light reaction, and complete or partial external paresis including ptosis with supra-, infra-, and adduction deficits [13].

Compressive lesions of oculomotor nerve usually affect both the central somatomotor fibers and the peripheral superomedial pupil fibers, while ischemic lesions spare the latter. This anatomy is the basis for the “rule of the pupil,” which states that a complete motor third-nerve palsy (complete external paresis) with a normal pupil is most probably ischemic in origin and not compressive [14].

When the peripheral oculomotor nerve is involved by an aneurysm, usually the pupilloconstrictor fibers are involved first, followed by palsy of the levator palpebrae, superior rectus, and medial rectus, in order [8]. In general, after both surgery and coiling, functional recovery is usually noted first in the levator palpebrae muscle, followed by the medial rectus muscle, superior rectus muscle, constrictor muscles of the iris, and ciliary muscle. Patients with incomplete recovery often had residual diplopia in upward gaze and pupillary dysfunction [15].

But in our case, the patient had reversal of ptosis occurring last in the order of recovery. In a large study on the prognosis of ocular motor nerve palsies conducted by Richards BW et al., it was concluded that the mean recovery time from ONP was 5.4 months and the range was from less than one month to 48 months. The median tended to be earlier, about 2.6 months. As might be expected, the more benign the cause, the more rapid the recovery [16]. In a study on cranial nerve lesions following aSAH, oculomotor nerve lesions regressed in 39.2% of cases within a period of six months; the remaining lesions were permanent [11]. But our patient had a relatively early recovery in two months.

There are only 12 properly reported cases of ONP associated with Acom artery complex aneurysms in the literature till date, including our case (Table 1). The initial reports of this rare phenomenon date back to as early as 1974, when ocular motor disturbances occurring as false localizing signs in ruptured intracranial aneurysms were reported by Suzuki J and Iwabuchi T [3]. The mean age of the patients was 58.7 and majority of the patients, including our case, were females (5:1). Most of them had a Fisher grade of 3 and Glasgow Coma Scale score of 15 at the time of admission. Six patients were hypertensive. Only three patients had partial ONP, whereas all others had complete ONP. The right side oculomotor nerve was affected in five patients and left side in another five, and bilateral oculomotor nerves were paralyzed in two patients. Even though 10 patients had Acom aneurysm, one patient had A1 aneurysm [3] and another one had ACA-A2 aneurysm [9]. Two patients had some other vascular anomaly [3, 5] and one had developed a pontine infarct [8]. Though average number of days to recovery from ONP was slightly higher (98.1 days), the median duration was 60.5 days. Among the reported cases, fastest complete recovery occurred in 24 days.

The probable causes by which ONP can occur postoperatively following surgical clipping of an aneurysm are

1. damage to the nerve during drilling of lesser wing of sphenoid [17];
2. uncal herniation due to brain edema or hematoma;
3. direct injury to the nerve or pressure effect;
4. aneurysm clip accidentally incorporating or applying pressure over the nerve;
5. the presence of an unrecognized aneurysm in the vicinity of third nerve, which was missed in angiogram;
6. instillation of papaverine over the surrounding blood vessels, as a preventive attempt against vasospasm [18];
7. development of cavernous sinus thrombosis;
8. brainstem infarction or hemorrhage.

Our case is unique in that this is the only case in which ONP occurred postoperatively following clipping of a ruptured Acom aneurysm, without any evidence of cisternal clot and direct or indirect injury to the nerve by any of the mechanisms described above. The hypothetical explanations to the development of ONP in our patient are as follows:

(1) Direct injury to the nerve produced neurapraxia by the jet of blood during intraoperative rupture of aneurysm.

(2) As a part of cerebral vasospasm, which may have affected the vasa nervorum of oculomotor nerve. Ipsilateral periorbital pain, as occurred in this patient following ONP, stands more in favor of vasospasm, as it can involve the sensory ganglia located within the nerve. But our patient had a Modified Fisher grade 1, which is reported to cause vasospasm only in 21.6% of cases [19].

(3) A right posterior communicating artery aneurysm, which was not able to be diagnosed with CT angiogram and postoperative Magnetic Resonance Angiogram, is a very rare possibility. Digital Subtraction Angiogram could not be done due to economic constrains. But the spontaneous resolution of ONP and of the pain associated with it stands against such a possibility. Also the order of recovery of ONP also is against it, since in our patient ptosis was the last to resolve, whereas it is the first to resolve in aneurysmal ONP [14].

## 4. Conclusion

ONP can very rarely occur following aSAH, even from aneurysms located away from the oculomotor nerve. If there are no features of raised intracranial pressure, there is not much to panic. Usual natural history is one of gradual recovery over a period of time, usually within a year.

## Figures and Tables

**Table 1:** Cases of oculomotor nerve palsy associated with subarachnoid hemorrhage from aneurysms at anterior communicating artery complex.

Sr No.	Author (Year)	Age/Sex^*∗*^	Fisher grade	GCS^†^	Day of appearance	Complete/ Partial	Side affected	Aneurysm size and projection	Day of recovery	Comments	Possible explanation
1	Suzuki J et al. (1974) [3]	59 / F	NA^‡^	NA	0	Partial	Right	Right A1^§^ 15 × 10 × 10 mm	24	Megadolichobasilar anomaly	Raised ICP^II^/ Vascular malformation

2	Suzuki J et al. (1974) [3]	48 / M	NA	15 (EVD)^¶^	2 (EVD)	Complete	Bilateral	3 mm (on autopsy)	Expired	None	Tentorial herniation

3	Coyne TJ et al. (1994) [4]	59 / F	3	14	0	Complete	Bilateral	NA	120	HTN^#^	Clot in cistern / raised ICP

4	Aiba et al. (2003) [5]	61 / F	3	15	0	Complete	Left	NA	30	Inverted left PCA and SCA*∗∗*	Unusual anatomy

5	Aiba et al. (2003) [5]	70 / F	3	Confused Delirious	0	Complete	Right	NA	60	None	Clot in cistern / raised ICP

6	Satyarthee et al. (2004) [6]	65 / F	3	15	0	Complete	Right	NA	180	HTN	Medial temporal haematoma

7	White JB et al. (2007) [7]	46 / M	3	15	0	Complete	Left	10 mm × 9 mm × 7 mm	NA	HTN	Clot / blood products

8	Kang SD et al. (2007) [8]	68 / F	3	Semicomatose	0	Complete	Left	NA	Partial – 1 year	Lacunar infarct of pons	Clot, herniation / vasospasm

9	Fairbanks C et al. (2011) [9]	? / F	3	15	0	Partial	Left	Superiorly and anteriorly measured 1×2×1 mm	30	ACA-A2*∗∗∗* aneurysm HTN	Mass effect, hemotoxicity and ischemia

10	Balossier A et al. (2012) [10]	55 / F	3	15	1 (Emb) *∗∗∗∗*	Complete	Right	NA	90	HTN	interpeduncular cistern haematoma

11	Srinivasan A et al. (2015) [2]	55 / F	3	9	0	Partial	Left	Antero-superior	Partial - 21	HTN	Perfusion deficits, hemorrhagic dissection of the nerve

12	Our case	60 / F	2	15	2 (PO)*∗∗* *∗∗∗*	Complete	Right	Antero-superior 8 mm × 7 mm ×5 mm	61	HTN	Neurapraxia by jet of blood or vasospasm

*∗* M: Male, F: Female; †: Glasgow Coma Scale score; ‡: details not available; §: A1 segment of anterior cerebral artery; II: intracranial pressure; ¶: following external ventricular drainage; #: systemic hypertension; *∗∗* PCA: posterior cerebral artery; SCA: superior cerebellar artery; *∗∗∗*ACA: anterior cerebral artery; A2: A2 segment of anterior cerebral artery; *∗∗∗∗*: following embolization; *∗∗∗∗∗*: postoperative.
